# A Narrative Review of Pathogenetic and Histopathologic Aspects, Epidemiology, Classification Systems, and Disease Outcome Measures in Systemic Sclerosis

**DOI:** 10.1007/s12016-022-08929-x

**Published:** 2022-03-07

**Authors:** Maria-Grazia Lazzaroni, Silvia Piantoni, Fabrizio Angeli, Stefania Bertocchi, Franco Franceschini, Paolo Airò

**Affiliations:** Rheumatology and Clinical Immunology Unit, Department of Clinical and Experimental Sciences, ASST Spedali Civili of Brescia, University of Brescia, Piazzale Spedali Civili 1, 25123 Brescia, Italy

**Keywords:** Systemic sclerosis, Vasculopathy, Epidemiology, Classification, Disease outcomes, Patient-reported outcomes

## Abstract

Systemic sclerosis (SSc) is a rare systemic autoimmune disease, characterized by the presence of three main actors: vasculopathy, immune activation, and fibrosis. This pathologic process is then translated in a clinical picture with great variability among different patients in terms of type of organ involvement, disease severity and prognosis. This heterogeneity is a main feature of SSc, which, in addition to the presence of early phases of the disease characterized by mild symptoms, can explain the high difficulty in establishing classification criteria, and in defining patients’ subsets and disease outcomes. The definition of disease outcomes is particularly relevant in the setting of clinical trials, where the aim is to provide reliable endpoints, able to measure the magnitude of the efficacy of a certain drug or intervention. For this reason, in the last years, increasing efforts have been done to design measures of disease activity, damage, severity, and response to treatment, often in the context of composite indexes. When considering disease outcomes, the experience of the patient represents a relevant and complementary aspect. The tools able to capture this experience, the patient-reported outcomes, have been increasingly used in the last years in clinical practice and in clinical trials, both as primary and secondary endpoints. This comprehensive narrative review on SSc will therefore cover pathogenetic and histopathologic aspects, epidemiology, classification systems, and disease outcome measures, in order to focus on issues that are relevant for clinical research and design of clinical trials.

## Introduction

Systemic sclerosis (SSc) is a rare systemic autoimmune disease, characterized by the presence of three main actors: vasculopathy, immune activation and fibrosis. Importantly, vasculopathy seems to play an early and crucial role in triggering the entire pathogenetic pathway [1].

The early phases of the disease are in fact characterized by the presence of Raynaud’s phenomena in virtually all patients, followed by the possible appearance of the “red flag” sign of puffy hands, and from a serological point of view by the presence of specific SSc autoantibodies [2]. Currently, the early phase of the disease is one of the main focuses of SSc clinical and translational research, representing a period of immune activation with potential higher sensitivity to immunosuppressive treatment.

During the disease course, the type of internal organ involvement is highly variable among different patients, with a very wide spectrum of severity, and consequently significant variations in prognosis in terms of both morbidity and mortality.

The presence of different temporal phases of the disease, characterized by different sensitivity to treatment, together with the significant heterogeneity and also the rarity of the disease, has increased the difficulty in establishing classification criteria and in the stratification of patients in different subsets, which is very relevant for both the clinical practice and the clinical trials. In this last setting, the necessity of defining the outcome of SSc patients in order to create reliable endpoints has led to an increasing effort in the development of objective measures to define disease activity, damage, severity, and response to treatment [3].

Importantly, the field of outcome measures in SSc not only is constituted by objective scores and indexes to quantify the magnitude of disease under different aspects, but is also complemented by patients’ perspective that can be systematically assessed through the patient-reported outcomes (PROs) [3].

Therefore, this narrative review will cover different topics, starting from pathogenetic and histopathologic aspects, and then dealing with epidemiology and classification systems, and also outcome measures including patients’ perspective through the patients’ reported outcomes. This comprehensive approach has the objective to review different aspects that are relevant for clinical research and design of new clinical trials in SSc, especially the stratification of patients to improve inclusion criteria, and disease outcome measures to better define endpoints, also including patients’ perspective through the patient-reported outcomes.

## Pathogenetic and Histopathologic Aspects in SSc

**Fig. 1:**
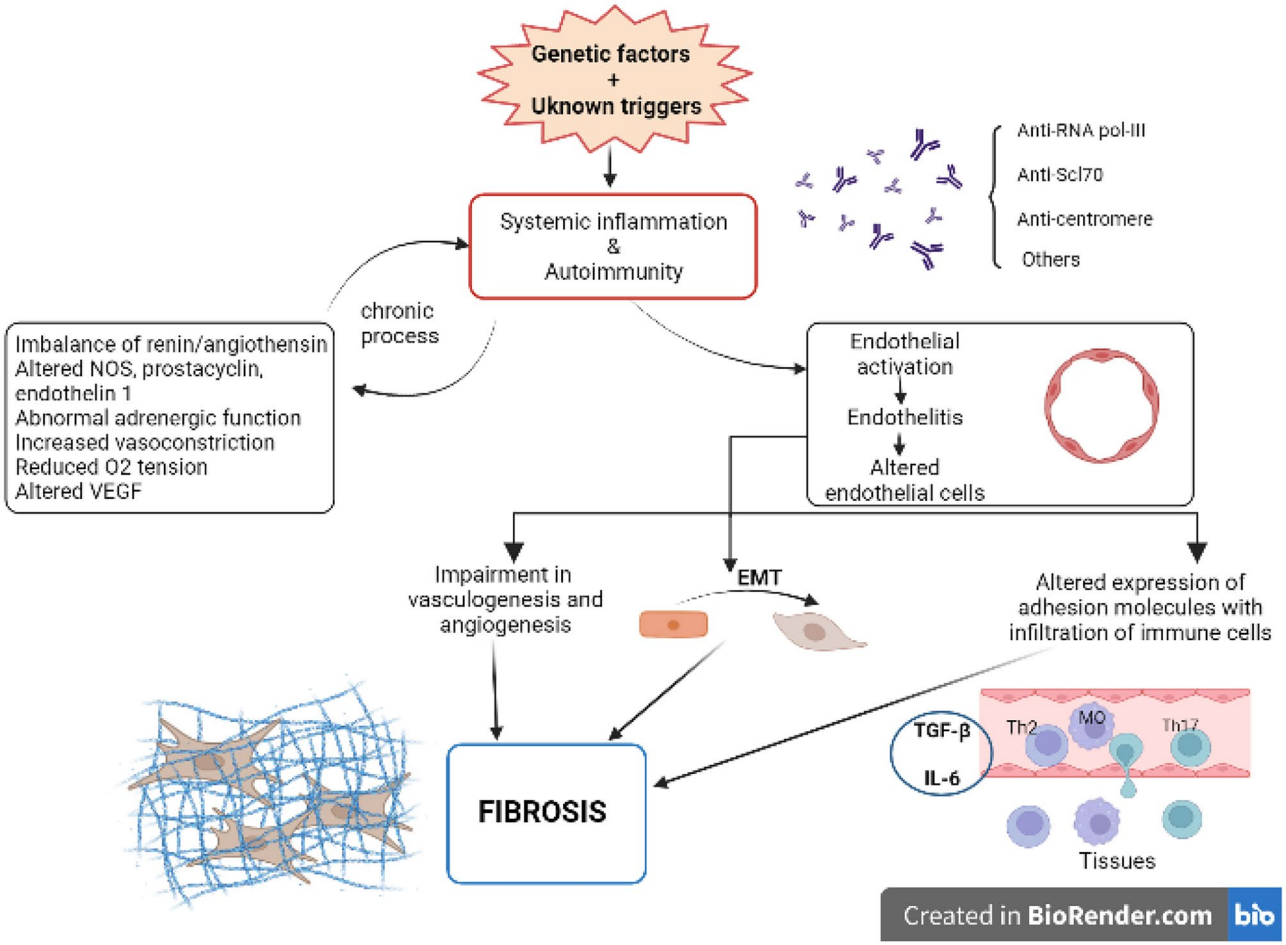
Overview on the pathogenesis of systemic sclerosis. Anti RNA-Pol-III, antibodies against RNA polymerase-III; Anti Scl-70, antibodies against scleroderma-associated autoantigen of 70-kDa; NOS, nitric oxide synthase; O2, oxygen; VEGF, vascular endothelial growth factor; EMT, endothelial-to-mesenchymal transition; TGFβ, tissue growth factor β; IL-6, interleukin 6; MO, macrophage; Th17, lymphocyte T helper-17; Th2, lymphocyte T helper-2. Created with BioRender. Academic license

The main pathogenetic mechanisms in SSc, which are shared across the multiple organ involvements, include vasculopathy, immune activation and fibrosis (Fig. 1). These aspects co-exist with additional organ-specific dysfunction [4]. Vascular injury, possibly due to autoimmune attacks triggered by unknown environmental factors, is the main event of a common pathologic cascade. Then, impaired angiogenesis and vasculogenesis promote vascular structural abnormalities [5], while endothelial cells with an altered expression of cell adhesion molecules enable the infiltration of circulating immune cells (mainly T helper (Th)-2 and Th17 cells, mast cells, and macrophages) into perivascular areas of various organs [6, 7]. Dysregulation of endothelial cells includes their activation with the induction of endothelial-to-mesenchymal transition leading to fibro-proliferative vascular change and tissue fibrosis, which becomes irreversible when chronic inflammation persistently activates interstitial fibroblasts [8]. Modifying factors in each organ, such as keratinocytes and adipocytes in the skin, esophageal stratified squamous epithelia and myenteric nerve system in gastrointestinal tract, vasospasm of arterioles in the heart and kidney, and micro-aspiration of gastric content in the lung, also have a role in organ-specific manifestations [4].

Vasculopathy, which is a bridge mechanism between immune alterations and fibrosis, is expressed at clinical level as Raynaud’s phenomenon (RP), digital ulcers (DU), scleroderma renal crisis (SRC), and pulmonary arterial hypertension (PAH).

RP, the most common clinical sign of the disease [1], found in more than 96% of SSc patients [9], is a clinical diagnosis indicating fingers’ color change, determined by an aberrant digital perfusion, which represents an exaggerated physiological response to trigger factors. Differently from the primary RP, SSc-related RP is an irreversible process which can result in substantial morbidity and disability, and, thus, is an important therapeutic challenge for rheumatologists [10]. During the episode, often caused by cold exposure or emotional stress, the initial pallor is caused by vasoconstriction of pre-capillary arterioles, then the following purple color is related to the cyanosis, and the final red color is caused by post-ischemic hyperemia (reperfusion) [10]. In SSc-related RP, the tissue ischemia is persistent and can result in DU and/or gangrene [11]. A central event in the pathogenesis of RP is the imbalance between vasodilatation and vasoconstriction, in favor of the latter. Despite the fact that mechanisms have to be fully elucidated, an impairment in vasodilatation, primarily due to an endothelial dysfunction, was well demonstrated [12], but other complex factors have been involved, such as the imbalance in the renin–angiotensin system, in favor of angiotensin II [11]. The alteration in the endothelial production of nitric oxide (NO), prostacyclin, and endothelin 1 is a matter of debate. Several studies demonstrated an upregulation of the inducible isoform of nitric oxide synthase (iNOS) in SSc, which leads to the production of large quantities of NO [13], that may have strong pro-inflammatory and cytotoxic properties [14]; on the other hand, the endothelial isoform of nitric oxide synthase (eNOS) is decreased [15]. Globally, the NOS function seems to be decreased in SSc endothelial cells, also for the decreased vasculature compliance due to the fibro-proliferative change [16]. In addition, reduced NO production via neuronal NOS (nNOS) could also have a role [17]. The significance of these mechanisms in SSc pathogenesis is demonstrated by the efficacious administration of drugs acting on NO signaling pathways, such as phosphodiesterase type 5 inhibitors and stimulators of soluble guanylate cyclase (riociguat). Structural abnormalities of the microvasculature (such as capillary dilatation and loss, and arteriolar stenosis, in the frame of progressive microangiopathy) and of the digital arteries contribute to impaired digital perfusion in SSc [18]. They represent a consequence of endothelial dysfunction (caused by endothelial injury, possibly autoantibody mediated, followed by endothelial cell apoptosis), aberrant production of growth factors and cytokines, activation of pericytes, and abnormalities of both angiogenesis and vasculogenesis [19]. As a contributing factor, an inhibitory splice variant of vascular endothelial growth factor (VEGF) leads to insufficient angiogenesis in patients with SSc [20].

Autonomic and small sensory nerve fibers of the vascular wall are also involved in SSc-related RP, favoring an increased vasoconstriction. Adrenergic function is abnormal in terms of hyper-responsiveness of alpha2 adrenergic receptor [21] and of increased protein tyrosine kinase activity and tyrosine phosphorylation [22]. Intravascular factors that lead to luminal occlusion and increased vasoconstriction, including platelet activation and impaired fibrinolysis, but also hypoxic–reperfusion injury and white blood cells activation, are relevant in SSc-related RP [23, 24]. More than half of SSc patients develops DU, which represent a major cause of morbidity and pain. This manifestation, which represents the underlying SSc vasculopathy and fibrosis, was reported to be present in 70% of SSc patients after 10 years of follow-up [25], with anti-topoisomerase I antibody (anti-Topo 1)–positive patients experiencing this complication at least 5 years earlier than those with anti-centromere antibodies (ACA) positivity [26]. This complication is driven by the persistence of ischemia and favored by recurrent microtrauma and increased skin tension [27], and has been associated with more severe disease phenotype, characterized by an early internal organ involvement [28]. Together with the microvasculature’s alterations as described above, an increased risk of DU is associated to a prevalence of macrovascular disease proximal to the digital artery, in particular affecting the ulnar artery [29]. DU can also develop in association with calcinosis, another common and debilitating manifestation of the disease [30]. The presence of infection, perilesional edema, necrosis, eschar, and gangrene are the major causes of a delayed process of healing [31]. It was reported that the about 40% of infected DU are associated with osteomyelitis, as defined by clinical and plain radiographic features, that may lead to amputation [31].

Regarding the histopathology aspects, the Masson’s trichrome staining of the digital arteries of SSc patients has revealed a fibrotic intimal hyperplasia, adventitial fibrosis with the alteration of arterial lumen [27]. Interestingly, these features can also be found in the arteries of the lung, kidney, and heart in SSc, but may differ in terms of frequency and severity according to different SSc subsets [27].

Another important manifestation of SSc vasculopathy is represented by SRC, which is a rare life-threatening complication with high morbidity and mortality [32]. It affects the 11% of diffuse and 4% of limited cutaneous SSc subjects [33]. Recent studies demonstrated that several factors could be considered predictive of this manifestation in SSc, such as chronic kidney disease, systemic arterial hypertension, and proteinuria [32]. Anti-RNA polymerase antibodies (especially types I and III) are significantly associated with the development of SRC, over the other important association with synchronous cancer [34, 35].

A broad spectrum of clinical manifestations has been reported, varying from a rapidly progressive renal insufficiency to modest renal dysfunction, with or without systemic arterial hypertension [36]. It has been reported that 40–50% of SRC cases can present signs of thrombotic microangiopathy (microangiopathic hemolytic anemia and/or thrombocytopenia), whose pathogenetic aspects still need to be fully elucidated [32]. Primary small vessel changes usually predominate over glomerular alterations in SRC, differently from what is found in hemolytic uremic syndrome, and thrombotic microangiopathy changes are more commonly detected in the glomeruli than in small vessels [37]. The intimal accumulation of myxoid material, thrombosis, and/or fibrinoid necrosis is found as the manifestation of vascular modifications. Acute glomerular changes occur often with the vascular injury and reduction in renal perfusion [38], while chronic glomerular alterations (e.g., glomerulosclerosis) may develop progressively. Tubulointerstitial modifications are represented by ischemic acute tubular necrosis or, if more chronic, as tubular atrophy and interstitial fibrosis [37].

Pulmonary hypertension (PH), another hallmark of SSc vasculopathy, is a hemodynamic condition defined by the presence of a mean pulmonary arterial pressure ≥ 25 mmHg evaluated by means of resting right heart catheterization [39]. It can represent a complication of left heart or lung diseases, which are common comorbidities in SSc patients, or a consequence of chronic thromboembolism, or a primary arteriolar vasculopathy (PAH). Inflammation and endothelial injury are precursors of this condition, whose manifestations can vary from an asymptomatic condition to a clinical picture characterized by progressive dyspnea, fatigue, and palpitations, as long as the pressure in the pulmonary vascular system progressively increases, leading to right-sided heart failure [40].

PAH in SSc is among the most frequent pulmonary vascular complication in SSc worsening significantly SSc patients’ prognosis [41]. In fact, it was reported that SSc–PAH patients have a 3-year survival prevalence of 61%, which is lower than that of patients with an idiopathic form (80%) [41–43]. The reason of this poor prognosis is mainly related to the intrinsic compromised right ventricle contractile function, due to fibrotic processes that involve the endocardium, with a consequent low grade of adaptive hypertrophy [44].

In summary, vasculopathy has a central role in the pathogenesis of SSc with a wide spectrum of clinical manifestations. Together with tissue fibrosis, it is a trigger of a common pathologic cascade that can be found across multiple organs and of the additional organ-specific pathology.

## SSc Classification

The history of SSc classification can be viewed as a combination of efforts of lumping (with the aim of increasing diagnostic sensitivity to include patients with milder forms of the disease, with the possible risk of increasing the heterogeneity among them) and splitting into subsets with different characteristics or prognosis.

The first set of classification criteria for SSc was published in 1980 by the American College of Rheumatology (ACR) [45]. These criteria were designed for classification of definite SSc, and not for diagnostic purpose, and, intentionally, to be specific rather than sensitive [45]. Briefly, they were based on the presence of a major criterion (scleroderma proximal to metacarpophalangeal joints), or 2 out of 3 minor criteria (sclerodactyly, digital pitting scars, and bilateral pulmonary fibrosis). Even if proposed as preliminary, these criteria remained in use for several decades, though it was widely recognized that they did not cover the full spectrum of patients suffering from SSc; in particular, the 1980 criteria had a good performance in advanced SSc, but lacked sensitivity for early forms and for limited cutaneous SSc (lcSSc) [46].

For these reasons, in 2001, LeRoy and Medsger proposed personal criteria for the classification of early SSc with the introduction of nailfold capillaroscopy and autoimmune serology abnormalities (which in the meanwhile proved to be reliable predictors of SSc) and Raynaud phenomenon (a symptom present in nearly all the patients with SSc) [47]. Moreover, they identified a group of patients as having “limited SSc without cutaneous involvement” (lSSc), defined as Raynaud phenomenon plus abnormal nailfold capillaroscopy and/or SSc selective autoantibodies. It was suggested that many of these patients had “early SSc” and might develop definite SSc during the follow-up [47]. The inclusion of this group of patients was a matter of debate, since some clinicians preferred to avoid overdiagnosis and suggested to not lump patients with subtle findings and patients with definite disease, using instead a term like “undifferentiated connective tissue disease (UCTD) with scleroderma features” for them [48]. Nevertheless, these criteria were frequently used (e.g., in some epidemiologic studies), even if they were not based upon a formal study. Noteworthy, some years later, a large prospective study of patients with Raynaud’s phenomenon demonstrated that the majority of the lSSc patients indeed develop definite SSc with additional clinical manifestations during the follow-up, particularly when both abnormal findings on nailfold capillaroscopy and SSc-specific autoantibodies were present [49], thus validating the LeRoy and Medsger criteria for early SSc.

The necessity of predicting which of these patients will develop SSc over time led investigators from the European Scleroderma Trial and Research (EUSTAR) group to propose “puffy swollen digits turning into sclerodactyly” as an adjunctive criteria for the very early diagnosis of SSc (VEDOSS) [2, 50].

Finally, a joint effort by the ACR and the European League Against Rheumatism (EULAR) led to the development of new consensus classification criteria that were published in 2013 [51]. Briefly, the 2013 criteria maintained the major criterion of proximal SSc from the 1980 set, and combined and integrated, with different weighted scores, the 3 minor criteria from the 1980 set, the items from the subsequent 2001 LeRoy and Medsger criteria, and 2 new items: telangiectasia and PAH [52].

Sensitivity and specificity of the new classification criteria in the validation cohort (half of them with less than 2 years of disease duration, in order to include early SSc) were 91% and 92% respectively that were significantly higher as compared to the 1980 or to the LeRoy and Medsger criteria.

The clinical utility of these new ACR/EULAR classification criteria for SSc was soon confirmed in independent cohorts of patients from different geographic areas. Generally, in these studies, unselected patients with a suspected diagnosis of SSc were included (e.g., patients with Raynaud’s phenomenon from a capillaroscopy clinic). In these setting, the sensitivity of the 2013 criteria was reported to range from 94 to 98% using the clinical judgment as gold standard, and performed better than the 1980 criteria [53–56]. However, the analysis of a real-life cohort with a particular focus on patients with mild/early disease showed that only 56% of patients not satisfying the 1980 criteria, but with an expert diagnosis of SSc, fulfilled the new 2013 criteria [57]. This group of patients had Raynaud’s phenomenon (93%), SSc pattern on nailfold capillaroscopy (73%), and/or SSc-specific antibodies (60%), but no sclerodactyly or other criteria, except for puffy fingers or telangiectasia in few cases. Some of these patients might have early disease (24% had disease duration < 2 years) and could progress to fulfill the 2013 criteria during the follow-up, but most of them had longer disease duration (median 6 years) and mild SSc [57]. The proportion of patients not satisfying the new 2013 criteria in a similar study from Japan was much lower (10%), but with similar features [58].

Indeed, these combinations of features resemble those cases identified as lSSc by LeRoy and Medsger or as VEDOSS, confirming the existence of a sizeable proportion of patients, not satisfying the 2013 classification criteria, with Raynaud’s phenomenon and good predictors of SSc (and, in some cases, clinical manifestations not included in the classification criteria, such as esophageal dysfunction) [59, 60]. While some experts would make a clinical diagnosis of SSc in these cases, others prefer to adopt a different definition (e.g., “UCTD at risk for SSc”) [61], considering that only a part of them will develop SSc over time, and are working to identify those with higher risk (labeled as “pre-scleroderma”) [62].

It should be noted that only the 3 more frequent SSc-specific antibodies (ACA, anti-Topo 1, anti-RNA polymerase III (anti-RNAP3)) are included in the 2013 classification criteria. Whether this might reduce the sensitivity of the criteria in patients with lSSc or lcSSc bearing rarer autoantibodies with high specificity for SSc (e.g., anti Th/To), or that may be associated with SSc (such as anti-PM/Scl), is unknown.

As mentioned above, the specificity of 2013 criteria is also high. It must be kept in mind that these criteria allow for classification of patients with another rheumatic disease as also having SSc, recognizing the possibility of overlap syndromes [51]; in particular, 10% of patients with mixed connective tissue disease (MCTD) were reported to meet the 2013 criteria [55], but it is likely that this figure was underestimated. Importantly, the classification should not be applied in patients without sclerodactyly, but with fibrotic skin involvement elsewhere (e.g., morphea) [63].

The 2013 criteria deliberately avoided the identification of subsets, because it was considered important to first classify a patient as having SSc before the assignment to different prognostic subsets. Nevertheless, the heterogeneity of SSc presentation and prognosis might be increased by the higher sensitivity of these criteria, reinforcing the need of identifying disease subsets with similar clinical manifestations and outcome and, possibly, similar pathogenesis [63].

## SSc Subsetting

### Cutaneous Subsets

The most common system of SSc subclassification, originally proposed in 1988 by LeRoy et al. is based on the extent of cutaneous involvement and identifies two subsets, defined as lcSSc or diffuse cutaneous SSc (dcSSc) [64]. In the original paper, other clinical features were also considered to be differently polarized between these two subsets (e.g., the time interval between the onset of Raynaud’s phenomenon and skin and internal organ involvement).

Other authors have proposed to use a 3-subset system based on the extension of skin involvement: digital (finger or toe), intermediate (proximal to metacarpophalangeal (MCP), but excluding trunk), diffuse (truncal sclerosis) [65, 66]. Although this classification might better reflect different prognostic groups, patients with intermediate SSc are heterogeneous regarding clinical features and autoantibody positivity (e.g., similar proportions of ACA + and anti-Topo 1 + patients) [67], and this system became less frequently used.

As mentioned above, patients without skin involvement can be classified as having SSc according to LeRoy and Medsger as well as to the ACR/EULAR 2013 criteria. Sometimes they are considered to represent a distinct subset (“lSSc,” or when typical SSc organ involvement is present, “sine scleroderma SSc”), but in most cases, in the current literature, these patients are included in the lcSSc subset.

### Autoantibodies Subsets

Although the two subset systems by Scussel-Lonzetti et al. have a fair discrimination between patients with different dominant pathogenetic features (vascular versus fibrotic), internal organ damage, and survival [67–70], it suffers from evident limitations. In common clinical practice, a certain degree of heterogeneity within these two clinical subsets can easily be observed: for example, around 1 of 4 patients with lcSSc patients has anti-topo I antibodies and, conversely, 1 of 3 anti-Topo 1 + patients has lcSSc [71]. Notably, in the large EUSTAR series, the risk of interstitial lung disease (ILD) in anti-Topo 1 + lcSSc patients was comparable to that of anti-Topo 1 + dcSSc; on the other hand, progression of ILD was instead slower, and the risk of other major complications were lower in anti-Topo 1 + lcSSc than in anti-Topo 1 + dcSSc [71].

These data highlight the role of autoantibodies (and their limitations) as predictors of organ involvement of SSc; a possible clinically easy-to-apply way to improve stratification of SSc patients might therefore take into account the combination of antibody profiles and skin subsetting of SSc [69, 72].

A good example of the results of this approach was presented by Nihtyanova et al.: by analyzing a large monocentric cohort of > 1,300 patients, they could identify seven SSc subtypes with different organ involvement and risk of death: ACA + lcSSc; anti-Topo 1 + lcSSc; anti-Topo 1 + dcSSc; anti-RNAP + ; anti-U3RNP + ; other antibodies lcSSc, other antibodies dcSSc [73]. Again, in this series, among anti-Topo 1 + lcSSc, a high frequency of ILD was observed, but other complications were rare, and survival was good. Patients with “other” autoantibodies and dcSSc had a poor prognosis and frequent organ complications, whereas lcSSc patients with “other” autoantibodies had low risk of kidney and heart involvement.

### Cluster Analysis Approach

A more sophisticated approach is based on the use of unsupervised ascendant hierarchical clustering of selected clinical and laboratory variables. For example, Sobanski et al. by evaluating the large series of patients included in the EUSTAR database, delineated two main clusters that indeed somehow overlapped with usual dcSSc or lcSSc prototypes [68]. In an exploratory attempt, 6 different clusters were also further characterized (albeit, with low reproducibility) that differed regarding their clinical features, autoantibody profile, and mortality. Some of them exhibited unique features, such as a cluster with a majority of lcSSc patients with a high rate of anti-Topo 1 antibodies, ILD, and other organ damage. The presence of organ damage markedly impacted survival, suggesting that beside cutaneous involvement, and antibody profile, organ damage should be taken into consideration when individuating homogeneous groups of patients with a distinct prognosis. A major limitation of this study was the lack of availability of autoantibody status other than ACA and anti-Topo. However, in a smaller monocentric study, it was shown that adding other autoantibody status to the cluster process resulted only in a partial contribute to risk stratification and clinical subsetting in SSc [69].

### New Approaches in SSc Subsetting

Finally, as discussed in another paper in this Journal, attempts to update SSc subsetting exploiting data deriving from new tools (e.g., transcriptomics or genomics) may lead in the next future to better identification of distinct clinical trajectories, improving personalization of SSc patient care [70, 74, 75]. Prior to implementation of new classification schemes, these will need to be proven superior to previous subsets, reliable, feasible, and valid [76].

## Epidemiology of SSc

In a recent meta-analysis, the overall pooled incidence rate of SSc was 1.4 (95% CI 1.1–1.9) per 100,000 person-years and the overall pooled prevalence of SSc was 17.6 (95% CI 15.1–20.5) per 100,000 individuals, but the incidence rate ranged from 0.2 to 7.5 per 100,000 person-years, and the prevalence ranged from 3.1 to 144.5 per 100,000 individuals [77]. This information highlights the great variability of data concerning the incidence and prevalence of SSc. There are several possible explanations for this variability.

### Design and Methodology of the Studies

A first explanation of this observed variability could be the existence of significant differences in the design and methods in different studies, concerning both case identification (data sources and case ascertainment) and definition [76–79]. In particular, although sometimes used also for prevalence and incidence evaluation, 1980 classification criteria suffered from poor sensitivity for early SSc and lcSSc, and were therefore not suitable to this task, inducing other groups [80, 81] to use also the classification criteria early SSc proposed by LeRoy and Medsger in 2001 [47]. Not surprisingly, the more recent ACR/EULAR 2013 criteria, which are more sensitive and useful also for early SSc patients classification, can identify more SSc cases, resulting in an around 40% higher estimate of SSc incidence and prevalence than the 1980 classification criteria [82, 83].

### Timeframe of the Studies

A second explanation is the timeframe in which the epidemiological studies were conducted may be another source of variability. Results from a meta-regression of prevalence against calendar period indicated that more recent studies reported higher incidence and prevalence estimate [77]. Accordingly, the prevalence of SSc estimated by a meta-analysis including only studies published between 2006 and 2016 was 23 cases per 100,000 individuals (95% CI: 16–29) [79], nominally higher than that above indicated [77]. In fact, several observational studies reported an increase in the SSc incidence over time [82, 84–86]. For example, in our experience, a continuous increase in SSc prevalence was observed in an Italian Alpine valley, during an 18-year-long period [82]. A 3-to-fourfold increase during this period was observed using either the 1980 or the 2013 criteria, but the proportion of patients with a SSc diagnosis not satisfying the 1980 criteria significantly increased with time. Using the 2013 criteria, the prevalence of SSc in this area at 31st December 2016 was 58.6 (95% CI: 44.8–76.6) per 100,000 persons with age over 14 years, and the incidence rate was 4.6 per 100,000 person-years (3.1–6.8) during the 2011–2016 period [82]. The increase in SSc incidence was accounted for by cases satisfying only the 2013 criteria, whereas the incidence of cases classified according to the 1980 criteria did not significantly increase [82]. Accordingly, patients recruited in recent years, compared to those observed during previous decades, had more frequently favorable clinical and prognostic features (e.g., limited cutaneous involvement and positivity for anticentromere antibodies). Therefore, it can be hypothesized that the rise of the SSc incidence and prevalence observed in the last years is due to more diffuse physician and patient awareness of the disease, and to the increased availability of diagnostic tools (nailfold videocapillaroscopy, SSc-specific autoantibodies), which may lead to wider recruitment of patients in the early stages of the disease [82, 86]. This might also explain the evolution of SSc pathomorphosis observed by some authors in recent years [86].

### Geographical Variability

A third reason for the variability in the observed incidence and prevalence could be related to geographical differences in SSc distribution that might also contribute to the variation of demographic parameters. Information on this issue from some large areas of the world (e.g., Africa and South America) is scanty [77]. The disease is more common in North America or Australia than in East Asia [77, 79]. In Europe, a north–south gradient with a higher prevalence in southern countries/regions was previously identified [78, 79], but recent data might challenge this view, describing a prevalence SSc in Northern Europe similar to that observed in Southern European regions [83, 87, 88], which is generally similar to that observed by studies from North America and Australia.

Geographical differences might be explained by ethnic factors, but limited information is available. The highest prevalence of SSc was reported in Choctaw Native American from Oklahoma (66 cases per 100,000 using the 1980 criteria over the 1990–1994 interval) [89]. In Alberta, Canada, the highest prevalence of SSc was also observed in the indigenous people, in which numerous diverse Tribal Nations were represented [90]. In USA, SSc prevalence is higher among African American women compared with European American women [91, 92]. In the African American population, the African ancestry-predominant HLA alleles were found to be associated with overall SSc risk, and with antifibrillarin antibody, a marker of more severe disease [93]. These observations may help to explain the increased frequency and severity of SSc among the African American population.

In fact, ethnic factors not only might influence the disease prevalence and incidence, but also are also associated with differences in SSc presentation, irrespectively of geographical location: Asian patients have a faster and earlier disease onset with high prevalence of pulmonary hypertension and lung function impairment and higher mortality than White patients, whereas Black patients have the fastest disease onset, a high prevalence of diffuse skin involvement and the highest mortality [94, 95]. These differences might not be caused exclusively by genetic factors. The role of socioeconomic factors in the explanation on the variability of SSc patients mortality among different ethnicities has been recently demonstrated in the USA [95] and will deserve future studies in other areas of the world.

Geographical differences in SSc-autoantibody distribution were also described, with highest frequency of anti-Topo 1 positivity in Asians, and highest rates of anti-Topo 1 positive and ACA negatives among Black [94]. Clearly, these differences might influence the clinical presentation. For example, a high degree of heterogeneity in the prevalence of anti-RNAP3 according to geographic distribution (lower in Asia and Southern and Central Europe; higher in Northern Europe, North America, and Australia) [96] might explain variability in the risk of anti-RNAP3-associated SSc manifestations, such as SRC.

Finally, environmental factors might account for some differences in SSc geographic distribution: exposure to organic solvents (e.g., aromatic or chlorinated compounds) [97] and heavy metals (e.g., antimony, cadmium, lead, and mercury) has been associated with SSc development [98]. Silica dust is the occupational or environmental risk factor most frequently identified in association with SSc [97, 99, 100]. Interestingly, a recent study showed significantly higher serum levels of silicone in SSc patients versus controls, particularly in patients with occupational exposure; higher levels of silicone were detected in patients with diffuse cutaneous SSc and/or lung fibrosis [100], thus reinforcing the hypothesis of a possible pathogenetic role of this element in the induction of SSc and its more severe clinical phenotypes. In this light, the much-debated possible link between silicone breast implants (SBI) and SSc might be reconsidered. It should be noted that a recent analysis of post approval studies by the United States Food and Drug Administration, including nearly 100,000 individuals with SBI, demonstrated an increased rate of SSc, as compared to normative data (standardized incidence ratio 7.00) [101]. Interestingly, studies from Japan and Italy demonstrated that, among SSc patients, SBI are associated with anti-RNAP3 [102, 103]. This association concerns particularly patients with documented SBI rupture, mostly without breast cancer [103]. These observations led to hypothesis that SBI rupture might elicit a particular autoimmune reaction against RNAP3 antigens, similar to what observed in some cases of breast cancer–associated SSc [34, 104, 105].

### Gender Influence in SSc

Gender differences in SSc have multiple implications, including differences in epidemiology, pathogenesis, and clinical expression of disease [106].

SSc is more common in females compared to males; the ratio between the genders varies in the literature between 3:1 and 14.5:1 [106]. However, it is more severe in males, which suffer more frequently from diffuse cutaneous and lung, heart, and kidney involvement [106, 107], whereas it is still not clear whether postmenopausal women are indeed particularly at risk for PAH, as it was previously suggested [106–108]. Moreover, there are great differences between genders in the association with autoantibodies: several studies indicated that men bear more frequently anti-Topo 1 and anti-RNAP3 or are ANA-negative than females, which in turn are more often ACA + , and, possibly, positive for anti-U3-RNP and anti-Th/To [106]. These observations might be explained by pathogenic sex differences. Much interest has been therefore dedicated to the X-chromosome; X-linked genes associated with SSc have been identified, and skewed X-chromosomal inactivation or epigenetic modifications of X-linked genes have been described in SSc [106]. The role of estrogens, particularly estradiol, is still debated [109]: these hormones were generally considered to have both a profibrotic and a vasodilatory effect (which may be protective against some SSc manifestation, such as PAH), but recently, an anti-fibrotic role of estrogens in pre-clinical models of SSc was demonstrated [110]. In a recent study [111], men with recent onset of dcSSc had higher levels of estradiol compared with healthy males and postmenopausal dcSSc. In these patients, high estradiol levels were associated with cardiac involvement and reduced survival. Besides genetic and hormonal factors, behavioral differences (e.g., smoking rates and occupational exposures) may contribute to different disease evolution between genders [106].

There is a great variability in the age of SSc onset, although SSc being more common in the middle age. It should be noted that disease severity might be influenced by the age of disease onset: patients with SSc onset in the elderly age more frequently suffer from lcSSc [112, 113]. Nevertheless, they suffer from an increased frequency of PAH, lung and heart involvement, and more rapid disease progression [113].

### Mortality in SSc

The mortality rate is still greater in SSc than in the general population. In a recent meta-analysis of 22 studies, the overall standardized mortality ratio (SMR) in SSc patients was 2.8 (95% CI 2.2–3.6) [114]. The SMR was very similar in European, North American, Asian, and Oceanian SSc populations. The SMR was numerically but not significantly higher in men than in women (3.5 (2.9–4.2) versus 2.9 (2.5–3.4)), whereas it was greatly higher in the dcSSc than in the lcSSc subset (4.9 (3.9–6.1) versus 2.0 (1.6–2.6)). Although some observations suggested a decrease mortality in SSc patients over time [86, 115, 116], this meta-analysis revealed only a not significant trend for a decrease in SMR with time [114].

More than half of the deaths in SSc patients are considered directly related to the disease [116, 117], and it has been observed that this rate gradually increased during the 2000–2011 period, suggesting that the possible increased survival observed in SSc might be explained more by the increased survival in the general population than by the improvement in SSc patient management [116].

Indeed, there was a well-documented change in the pattern of SSc-related deaths in the 1972–2002 period, in which the frequency of deaths due to SRC significantly decreased, possibly as a result of improvement in the management of this complication, whereas the proportion of patients with scleroderma dying of ILD or pulmonary hypertension (PH) increased [115]. In fact, ILD and PH were the leading causes of SSc-related deaths in the last two decades [116, 117] and future studies will clarify whether improvement in their management will change this picture. On the other hand, primary heart involvement (mainly, heart failure and arrhythmias) is another leading cause of death directly related to SSc [116, 117], indicating the need for a prompt and systematic management of this still often underrecognized SSc-associated complication. A meta-analysis of prognostic factors identified age at disease onset, male gender, African origin, dcSSc, anti-Topo 1 antibodies, cardiac and renal involvement, ILD, PH, and malignancy as associated with a worse prognosis [118].

A reliable prognostic score would represent a helpful tool for the clinician in the identification of poor-prognosis patients, who might benefit from aggressive therapy. In patients with dcSSc and disease duration of less than 15 months, skin fibrosis progression within the first year of observation is associated with worsened survival and represents a good predictor [119], even if it is useful only for a minority of SSc patients in everyday clinical practice. A simple prognostic model to predict 5-year survival in SSc including age, gender, proteinuria, high erythrocyte sedimentation rate (ESR), and low carbon monoxide diffusing capacity (DLCO) was developed by Bryan et al. in 1999 in 280 patients [120], and it was thereafter validated in a European multicentre study [121]. Using data from a sample of over 11,000 patients from the EUSTAR database, Elhai et al. developed a simple-to-calculate score (named the SCOpE score) that was a good predictor of 3-year all-cause mortality (AUC: 0.82) (Table 1) [116]. The 3-year survival in 4 different groups classified according to increasing scores was respectively 0.98 (0.97–0.99) (score 0–4), 0.93 (0.92–0.94) (score 5–9), 0.80 (0.78–0.83) (score 10–14), and 0.53 (0.48–0.58) (score ≥ 15). [116]. However, the authors were not able to externally validate the final model, though using the bootstrapping method as a validation tool.

**Table 1:** The SCOpE score: predictors of 3-years mortality in SSc patients derived from the EUSTAR cohort

**Item**	**Score**
Age 50–65 years	3
Age > 65 years	6
Male sex	1
Diffuse cutaneous disease	1
Scleroderma renal crisis	2
Prominent dyspnea	3
Digital ulcers	1
Contracture	1
Muscle weakness	1
Elevated C reactive protein	4
Proteinuria	3
Left ventricular ejection fraction < 50%	2
Interstitial lung disease	1
Carbon monoxide diffusion capacity < 60% predicted	4
Forced vital capacity < 70% predicted	2

SCOpE score ranges from 0 to 32 and allows to dive SSc patients into four groups according to the risk of mortality: low (score 0–4), low-intermediate (score 5–9), high-intermediate (score 10–14), high (score ≥ 15)

From: Elhai et al. [116]

## Outcome Measures in Systemic Sclerosis

In the last decade, the concept of “treat-to-target” has been increasingly used across different rheumatic diseases, demonstrating an association of a remission or low disease activity status (indicated as targets) with decreased morbidity and mortality. Currently, the “treat-to-target” paradigm still represents an elusive objective in SSc both in clinical trials and in everyday practice, for different reasons. First, there is a lack of effective “disease modifying anti-rheumatic drugs” in SSc, able to significantly modify disease course. Second, the disease is multifaced with variable organ involvement, thus increasing the difficulty in defining the status representing the target to be reached. In the absence of validated comprehensive disease measures, historically, the endpoints have been set on an organ basis, with skin involvement measured by modified Rodnan skin score (mRSS) as the most frequently selected in trials regarding dcSSc [122].

Besides the high variability in the type and severity of clinical manifestations among different patients, another issue in SSc is the presence of different phases of the disease in the same patient, so that timing is another variable to consider. In fact, the acquisition that an early phase of the disease, with more “inflammatory” phenotype, could be the one with the highest chance of treatment response, has significantly changed the inclusion criteria of clinical trials, which are now mainly targeting patients with a recent disease onset, especially early dcSSc [123].

The lack of definite outcome measures in SSc has been frequently advocated as potentially responsible for the failure of many clinical trials. In the last years, several initiatives from leading experts in the field of SSc have been undertaken to resolve this issue, such as the publication in 2014 of the “22 points to consider for clinical trials in Systemic Sclerosis, based on EULAR standards” [124].

Moreover, to face the necessity of standardized outcome measures, in 2008, the Scleroderma Clinical Trials Consortium (SCTC) working under Outcome Measures in Rheumatology (OMERACT) proposed a comprehensive set of 11 core domains (skin, musculoskeletal, cardiac, pulmonary, renal, gastrointestinal, Raynaud’s phenomenon, digital ulcers, global health, health-related quality of life [HRQoL] and function, and biomarkers) and 31 clinical measures to be applied in trials involving SSc patients [125, 126]. Interestingly, a recent review published in 2020 analyzed 152 trials in SSc including 4,193 outcomes classified into 84 domains, and found that none of these trials reported the complete core set with adherence to all the 11 SCTC core domains, with the majority of the studies reporting only 0–3 of them. The 3 domains most commonly recorded in the included trials were HRQoL and function (59%), skin (47%), and pulmonary (45%), with a very high number of different measures used (130, 59, and 168 different measures respectively) with high variability in the method of aggregation and metric. For example, regarding the skin, the validated mRSS or its variations were reported in 37 different ways [127].

### Composite Outcomes in SSc

On the basis of the abovementioned SCTC core set, afterward, the provisional Combined Response Index in Systemic Sclerosis (CRISS) was developed and approved by the ACR, with the objective of improving outcome assessment in clinical trials. It is composed by 2 clinical measures (mRSS, physician global assessment (PhGA)), 2 patient-reported measures (patient global assessment (PGA) and Health Assessment Questionnaire (HAQ)), and 1 surrogate measure (% predicted-forced vital capacity (FVC%)), to be used in randomized trials for patients with dcSSc [128, 129]. Given its nature of weighted score, it can be difficult to interpret and here is concern that it can be driven by one core set measure, especially mRSS, since it has the highest coefficient. Moreover, recent data from lenabasum phase III trial and a phase II trial of autotaxin inhibitor suggested a significant floor and ceiling effect of ACR-CRISS, although it should be noted that both trials allowed background immunosuppressive therapy in both the arms (new drug versus placebo).

This tool assesses the likelihood of improvement after 1 year, with a cut-off score of ≥ 0.6 considered the most sensitive and specific for improvement. It is calculated with a 2-step process, where step 1 identifies patients with significant worsening or new end-organ damage, which are assigned with a score of 0. Step 2 estimates the likelihood of improvement after 1 year, using the CRISS equation.

After its publication, CRISS has been used in clinical trials, but is still lacking external validation outside this setting, even if it was originally derived by observational cohorts.

For this purpose, the Canadian Scleroderma Research Group (CSRG) recently coordinated a collaborative study addressing the agreement between CRISS definitions for improvement and physicians’ evaluation of the disease. Specifically, 100 patients with characteristics similar to the original derivation cohort for CRISS (dcSSc and disease duration < 5 years) were randomly selected from a large observational cohort, including 50 with improved and 50 with non-improved CRISS (defined as dichotomous variable, with score ≥ 0.6 corresponding to improvement). Patients’ profile with a short report of their clinical history over 1 year was evaluated by a panel of 15 multinational experts, so that one patient was independently rated by 3 different physicians. A substantial agreement was observed between both physician majority opinion and each individual physician opinion, and CRISS, although the last tended to rate more patients as improved than physician [130].

Another study from the CSRG used the CRISS as the outcome to retrospectively assess the outcome of dcSSc patients exposed to immunosuppressive therapy in a multicentre cohort and found that 47 patients newly exposed to immunosuppression ≥ 1 year, as compared to 254 unexposed patients, had higher frequency of CRISS improvement (score ≥ 0.6 at 1 year). Interestingly, among the individual variables included in the CRISS, only PGA scores were significantly better in exposed than in unexposed patients. Since the possibility of a bias represented by a placebo effect affecting PGA improvement was excluded through a post hoc analysis, the authors discussed that this discrepancy could reflect the presence of aspects not explored by the objective measures included in CRISS, such as pain, fatigue, and other organ involvements. They concluded that altogether, these results suggest that a composite score within an individual could represent a more sensitive and comprehensive measure than the aggregate mean of organ-specific measures across individuals [131].

In order to improve these potential limits of the provisional ACR-CRISS, in 2020, Khanna et al. proposed a revised version, developed on a retrospective analysis on a pooled cohort of 354 SSc patients derived by 3 clinical trials (phase II ASSET trial, abatacept versus placebo; phase II and phase III trials on tocilizumab versus placebo) [132]. Two thirds of the participants were randomly selected to constitute the development sets (*n* = 237) and the remaining one third of participants formed the validation sets. The five domains of the CRISS were evaluated in this cohort in order to assess whether a certain percentage of improvement, in a certain number of domains (similarly to ACR20 response criteria for rheumatoid arthritis) could differentiate the effect of the active medication group from the placebo group. They observed a significantly higher proportion of improved patients in the active arm than in the placebo arm, both using the criteria of  ≥ 20% improvement in ≥ 3 core sets, but also using different cut points and number of domains. Specifically, the effect was consistent from 10 to 60% improvement in ≥ 1 core set measure. Finally, the authors proposed to consider ACR-CRISS20 or 25%, which translates into at least 20% or 25% improvement in mRSS, Health Assessment Questionnaire Disability Index (HAQ-DI), Patient Global Assessment and Physician Global Assessment, with 5% or 10% improvement in FVC. This is based on the minimal clinically important differences (MCIDs) that are published in different rheumatic diseases, including SSc.

The concept of a composite score has also been address in the SCOT trial assessing the efficacy of myeloablative autologous hematopoietic stem cell transplantation (HSCT) in SSc [133]. In fact, the primary endpoint that was reached at 54 months was settled on the Global Rank Composite Score (GRCS) that is an analytic tool including multiple disease manifestations simultaneously, but not measuring disease activity or severity. It is based on a hierarchy of ordered outcomes: death, event-free survival, FVC%, HAQ-DI, mRSS [134].

The other randomized trial assessing the efficacy of non-myeloablative HSCT in SSc, the French ASTIS trial [135], had similar inclusion criteria, but different conditioning regimen and different primary endpoint (24-month event-free survival). Interestingly, a very recent study assessed the long-term outcome of patients included in this trial, but using GRCS (as in the SCOT trial) and confirmed its superiority at 60 months as compared to cyclophosphamide, thus supporting the use of this tool in future trials in SSc [136].

## Disease Activity in SSc

The definition of disease activity as the component of disease severity that is largely reversible is particularly challenging in SSc, as disease course is significantly different from other connective tissue diseases (CTDs), such as systemic lupus erythematosus (SLE), in which on–off phases are more clearly identified. Accordingly, a low disease activity status, possibly associated with better long-term outcomes of morbidity and mortality and HRQoL, still has to be defined. Recently, Nagaraja et al. provided a preliminary proposal of definition of low disease activity status, influenced by data obtained from RCTs and observational studies, that will need rigorous testing and validation using a consensus methodology in future studies (Table 2) [137].

**Table 2:** Suggested parameters for defining low disease activity state in SSc on or off pharmacologic therapy

**Item**	**Criteria**	**Number of criteria needed**
**Skin in moderate-to-severe dcSSc**	mRSS ≤ 10 units	All 3
	HAQ-DI ≤ 0.75 units	
	PGA ≤ 3 units (on a 0–10 scale)	
**Established moderate-to-severe ILD**	FVC ≥ 70%	All 3
	Stable fibrosis and total lung involvement based either on visual read by a radiologist or by computer quantification	
	No worsening of dyspnea related to ILD	
**Raynaud’s phenomenon**	Mean RCS score ≤ 2/10	≥ 2
	RP attack frequency of ≤ 7/week (on a 0–10 scale)	
	Mean aggregate daily duration of RP attacks ≤ 15 min	
**Digital ulcers**	≤ 1 active digital ulcers in the past 6 months	All 3
	Low digital ulcers pain scale (≤ 3) on a 0–10 scale	
	Low SHAQ digital ulcer sub-scale (≤ 3) on a 0–10 scale	
**Scleroderma renal crisis**	Stable blood pressure on anti-hypertensive therapy	All 3
	Serum creatinine within 10% from pre-SRC serum creatinine	
	Transient to no requirement of hemodialysis	
**Moderate-to-severe PAH**	**Modified ESC/ERS (analyzed at time of RHC or first follow-up visit)**	≥ 3
	NYHA class I/II	
	6MWD > 440 m	
	RAP < 8 mmHg	
	CI ≥ 2.5 L/m/m^2^	
	**or**	
	NYHA class I/II	≥ 2 (non-invasive measures)
	6MWD > 440 m	
	BNP of 50 pg/mL or NT-proBNP < 300 pg/mL	
	**or**	
	**REVEAL 2.0 risk score ≤ 8 (low to intermediate**)	

*dcSSc* diffuse cutaneous SSc, *mRSS* modified Rodnan skin score, *HAQ-DI* Health Assessment Questionnaire Disability Index, *PGA* patient global assessment of disease activity, *ILD* interstitial lung disease, *FVC* forced vital capacity (percent predicted), *RCS* Raynaud’s Condition Score, *RP* Raynaud’s phenomenon, *VAS* visual analog scale, *SHAQ* Scleroderma Health Assessment Questionnaire, *SRC* scleroderma renal crisis, *PAH* pulmonary arterial hypertension, *ESC/ERS* European Society of Cardiology and European Respiratory Society, *NYHA* New York Heart Association, *6MWD* 6-min walking distance, *RAP* right atrial pressure, *CI* cardiac index, *BNP* B-type natriuretic peptide, *NT-proBNP* N-terminal proBNP, *REVEAL* Registry to Evaluate Early and Long-Term PAH Disease Management

From: Nagaraja et al. [137]

### EUSTAR Activity Index (EScSG-AI)

Currently, the only existing disease activity index in SSc is the EUSTAR Activity Index (EScSG-AI) that was published in 2001 and subsequently updated in 2016. The updated version consists of a 10-point weighted score of 6 items: patient-reported skin worsening over the preceding month, digital ulcers, absolute mRSS, tendon friction rubs, C-reactive protein (CRP) > 1 mg/dL, DLCO < 70% predicted. It was developed from the multicentre EUSTAR cohort, and included items identifying disease manifestations correlated with experts’ assessment of disease activity. This revised version has only been partially validated in a cohort within the EUSTAR registry: when a score ≥ 3 identifying active disease was compared to experts’ assessment of disease activity, the sensitivity was 52.2% and the specificity was 89.1%. Important concerns have been raised about the face and the content validity of this index, since it does not include gastrointestinal or renal activity, and in the revised version, there are only limited measures of cardiopulmonary involvement. Moreover, the use of absolute values of mRSS and DLCO has been criticized, as it may better reflect damage than activity [138, 139].

Recently, the original EScSG-AI was compared with the revised version, with the specific aim to evaluate their ability to detect dcSSc patients requiring treatment intensification in a longitudinal monocentric Belgian cohort of 62 patients [140]. The authors used a pragmatic definition of disease progression, which included “any start or increase of glucocorticoids, immunosuppressants, anti-endothelin receptors or prostanoids.” Both the scores were proved to be predictive of disease activity, with a slight better sensitivity for the revised version. Interestingly, patients with an active disease according to the original EScSG-AI had a 73% chance of effectively suffering from disease progression requiring step-up therapy, while this dropped to 59% for the revised version, thus indicating that this version could lack specificity for this purpose, possibly leading to overtreatment.

## Organ Damage in SSc

Damage has been defined as “the permanent and irreversible loss of anatomical structure or physiological function, caused by SSc and not secondary to its treatment or comorbidities” [141].

### SCTC Damage Index

In 2019, the SCTC was the first to develop a damage index in SSc (SCTC-DI). It was developed through a consensus strategy plus a statistical analysis on patients’ data [141]. In fact, the first step consisted of a web-based survey proposed to 331 SCTC members in order to evaluate the appropriateness of each item for inclusion; the items that obtained > 60% of consensus were retained. Finally, 93 of 331 members (28.1%) fulfilled the survey and 58 out of 83 proposed items were retained.

The second step consisted of statistical analyses performed to evaluate the association of these items with endpoints of morbidity (specifically Physical Component Summary score of the Short Form 36) and mortality. The first was a univariable analysis on a prospectively acquired cohort, in which 22 out of 58 items resulted significantly associated and were entered in a multivariable model, together with one additional outcome (“SRC and persistent renal impairment”), that due to its rarity did not reach the statistical significancy but was considered very relevant by the working group. The 23 items were included in a multivariable regression analysis in order to obtain the coefficients to create a 23-item weighted score, divided into 6 organ domains, that was named SCTC damage index (SCTC-DI) (Table 3) [141]. The authors also distinguished 3 levels of damage: 0–4 low, 5–12 moderate, > 12 severe.

**Table 3:** Scleroderma Clinical Trials Consortium (SCTC) Damage Index

**Item**	**Score**
**Musculoskeletal and skin**	
Joint contracture defined as any degree of contracture with the inability to reduce the joint to the anatomically neutral position in any small joint of the fingers*	2
Joint contracture defined as any degree of contracture with the inability to reduce the joint to the anatomically neutral position in the large joints, specifically elbows and knees*	2
Sicca symptoms defined as presence of dry eyes and/or dry mouth requiring treatment on a daily basis, for example, lubricant eye-drops, punctual plugs, saliva replacement*	3
Proximal muscle weakness on clinical examination defined as shoulder abduction and/or hip or knee flexion less than 5/5 power (not due to contracture or pain)*	3
Calcinosis complicated by infection or requiring surgery	4
**Vascular**	
Digital ulceration defined as loss of epithelialisation, of any degree, of the epidermis, the dermis, and/or the subcutaneous tissue, distal to or at the proximal interphalangeal joint of the hands or feet not thought to be due to trauma and refractory to therapy*	2
Add 1 if digital amputation required (surgical or autoamputation)	1
**Gastrointestinal**	
Oesophageal dysmotility defined as distal dysphagia refractory to treatment, with differential diagnoses (e.g., oesophageal stricture or malignancy) excluded by endoscopy	1
Oesophageal stricture confirmed on testing such as endoscopy or barium swallow	1
Symptoms of gastro-oesophageal reflux disease (heart burn) refractory to treatment (e.g., proton pump inhibitors) and confirmed on endoscopy*	1
Gastric antral vascular ectasia confirmed on endoscopy	2
Pseudo-obstruction with symptoms such as vomiting or constipation, with dilatation of the small and/or large bowel on imaging	3
Low body mass index of < 18.5 kg/m^2^ or weight loss of > 10% in the last 12 months	2
**Respiratory**	
Moderate to severe interstitial lung disease > 20% extent on HRCT of the chest	2
Add 4 points if forced vital capacity < 70% on lung function tests (not due to respiratory muscle weakness)*	4
Dependence on home oxygen	5
**Cardiovascular**	
Pulmonary arterial hypertension (defined as mean pulmonary arterial pressure > 25 mm Hg at rest and pulmonary arterial wedge pressure < 15 mm Hg on right heart catheterisation)	2
Add 5 if moderate to severe right ventricular dysfunction noted on echocardiography report based on assessment of any measure of RV function by experienced cardiologist	5
Myocardial disease attributable to SSc based on a constellation of clinical features and supportive investigations, for example, syncope secondary to conduction abnormality, arrhythmia requiring defibrillator, heartblock requiring permanent pacemaker or ablation, systolic or diastolic dysfunction on TTE	3
Presence of moderate to large pericardial effusion equivalent to greater than 1 cm on TTE*	1
**Renal**	
History of scleroderma renal crisis (SRC), either hypertensive or normotensive, as defined by the International Scleroderma Renal Crisis Study Investigators	3
Add 1 if history of SRC or other SSc-related kidney disease and persistent renal impairment with estimated glomerular filtration rate < 45 mL/min/1.73 m^2^	1
Add 2 if SRC with stage 5 renal impairment and need for renal replacement therapy	2
**Total score**	**55**

Attribution to SSc required for all items

*HRCT* high-resolution CT, *RV* right ventricular, *SSc* systemic sclerosis, *TTE* transthoracic echocardiogram

*Item must be present for a minimum of 6 months

From: Ferdowsi et al. [141]

Finally, SCTC-DI was evaluated in an external Canadian validation cohort, in which it was confirmed to be predictive of morbidity and mortality.

Noteworthy, the score is not cumulative as it happens in damage indexes used for other CTDs, such as SLE, because some of the items finally included are actually reversible disease manifestations, and this also represented the main issue raised after its publication [142].

Nevertheless, in both the cohorts considered in the original study above mentioned, and in a large single-center retrospective observational cohort from Italy of 253 SSc patients with a complete 10-year follow-up, the SCTC-DI score was shown to have a progressive significant increase over time [143]. Moreover, the proportion of patients with moderate and severe damage also increased over time. Particularly, in the retrospective Italian cohort, the proportion of patients with moderate and severe damage (DI score > 4) increased from 9% at the baseline (corresponding to the time of SSc diagnosis) to 34% at 10-year follow-up. Interestingly, the presence of dcSSc was associated with the presence of SCTC-DI > 4 at 5 and 10 years of follow-up, while ACA positivity was negatively associated at 5 years but not at 10 years. In the same cohort, SCTC-DI was also confirmed to be predictive of mortality [143].

## Disease Severity in SSc

Severity of SSc has been defined as a combination of both activity and damage. Currently, the only existing measure of severity in SSc is represented by the Medsger Severity Scale (MSS) that was created combining reversible and irreversible components of the disease [144]. It was constructed by consensus plus data-driven methods, using the Pittsburgh SSc cohort as a derivation cohort. Importantly, it was developed with the intent to represent the total effect of the disease on organ function, with a score of 0–4 for severity (no, mild, moderate, severe, end stage) in each one of 9 organ systems (general, peripheral vascular, skin, joint/tendon, muscle, GI tract, lung, heart, kidney).

Importantly, the authors specifically stated that the MSS would have been used as 9 separate scores, and not as a composite score, since they felt that this could misrepresent the overall severity of the disease, although a simple summed score of the original 9 domains or modified versions of the MSS scores have been used in subsequent studies, although without validation [145–147].

In a Swedish cohort of 100 SSc patients followed over 14 years, higher MSS scores (although not all the original items were assessed) were demonstrated to be predictive of higher rates of mortality [148]. Notably, the primary determinants of mortality were extensive skin involvement, ECG changes, and impaired lung and renal function.

More recently, Harel et al. [149] performed a cross-sectional study on a multicentre observational cohort of 875 SSc patients with the objective to develop a weighted summary version of MSS, and to compare it with the simple summed version of MSS and with the PGA of disease severity. The weighted MSS was derived through a statistical model on the abovementioned cohort, and attributed different weights (coefficients) to the 9 different domains of MSS. The results showed the highest coefficient for skin involvement (2.47), while a coefficient around 1 was reported for general system, joint/tendon, GI, and muscle, and unexpected low coefficients were found (below 1) for peripheral vascular, heart, lung, and kidney scales. Interestingly, the newly developed weighted MSS, the simple summed MSS, and PGA showed similar convergent and discriminative validity for the patient-reported outcomes considered, and also had similar predictive ability for mortality. Moreover, the inter-physician heterogeneity, measured as intraclass correlation coefficient (ICC), was higher for PGA, although without significant differences, meaning that there was no substantial contribution of the subjective component in the 3 measures.

In conclusion, all the 3 scores considered in this study appeared valid and performed similarly, but the concerns raised about the unexpected low weights of the lung, heart, and kidney led the authors to consider the PGA as the current preferred measure, encouraging further work to improve MSS.

## Patient-Reported Outcomes

SSc is associated with a significant impairment in physical functioning and psychological well-being, determining a significant reduction in HRQoL. In fact, the impact of the disease on different aspects of everyday life is an experience only known to the patient, and patient-reported outcomes (PROs) represent the tools able to systematically assess and quantify this aspect. Therefore, PROs have been increasingly used in clinical practice, for example, to guide and complement decision systemic treatment [150].

Recently, a paper from the Australian Scleroderma Cohort Study examined 1,636 SSc patients and found a significant association between patient-reported symptoms and changes in disease activity over time, measured as changes in different objective items (mRSS for skin involvement, pulmonary function test (PFT) parameters for ILD) or as the new onset of cardio-pulmonary involvement (ILD, PAH) [151]. These results underline the importance of patients’ perspective in everyday clinical practice, and its possible role as a substitute of objective measures when unavailable.

Moreover, PROs have also been increasingly used in clinical trials for SSc, either as primary or as secondary endpoints, in some cases as components of composite SSc outcome measures, such as the CRISS [129] or the GRCS [133].

### General PROs

Several existing PROs assess HRQoL in general, evaluating the impact of the disease on physical and mental function and on daily activities, and, even if not specifically developed for SSc, have demonstrated validity in different studies. Among these questionnaires, the most frequently used include the HAQ-DI, the Medical Outcome Study Form-36 (SF-36), and Patient-Reported Outcomes Measurement Information System-29 (PROMIS-29), which have also demonstrated a strong inter-PRO correlation.

The SHAQ (scleroderma-HAQ) has been developed by adding 7 SSc-specific domains to the HAQ-DI, which are scored with a Visual Analogic Scale (VAS) ranging from 0 to 10, able to explore different disease manifestations. Specifically, the 7 VAS domains include pain, general function, arthritis, gastrointestinal involvement, dyspnea, Raynaud’s phenomena, and digital ulcers [152].

Recently, the EUSTAR group provided a very interesting insight on the value of HAQ-DI in predicting the long-term outcome of dcSSc patients [153]. In fact, they performed an observational longitudinal study on 690 dcSSc patients registered in the EUSTAR database with at least one HAQ-DI score available, and demonstrated through a multivariable analysis that baseline HAQ-DI score and major advanced organ involvement had the same value in predicting mortality. Moreover, a sub-analysis on 424 patients who had at least 2 HAQ-DI scores available showed that baseline mRSS and baseline HAQ-DI score were predictive of HAQ-DI score progression at 1 year, thus highlighting a correlation between these endpoints in monitoring disease progression.

Another study from the EUSTAR network explored functional disability and its predictors within a specific EUSTAR prospective cohort, called the “DeSScipher cohort” [154]. Among 944 patients from this cohort who had complete SHAQ and HAQ scores, 40% had moderate or severe SHAQ (score ≥ 1). Interestingly, in the multivariable regression analysis, the main factors associated with high SHAQ scores were dyspnea, muscle weakness, digital ulcers, and gastrointestinal systems, indicating that certain types of organ involvements are perceived as more disabling by the patients, highlighting potential differences between the patients’ experience and the factors considered by the clinicians to guide the therapeutic pathway.

### Organ-Specific PROs

The experience of SSc patients regarding specific organ involvements can be captured by targeted PROs.

As an example, the UCLA SCTC GIT 2.0 is a PRO able to capture the magnitude of gastrointestinal manifestations, which has been specifically developed for SSc [155]. It includes 34 item and 7 multi-item scales and a total GIT score and was demonstrated to be reliable and feasible.

Very recently, Sibeoni et al. have published a newly developed PRO for a comprehensive evaluation of hand involvement in SSc, called the HAnDE Scale [156]. It is composed of 16 items divided in 5 levels of answers (range 0–64) and importantly, it was developed and validated only in SSc patients, assessing multidimensional aspects of hand involvement: functional, aesthetic, relational, existential, and emotional. It also showed a significant correlation with many existing general PROs, including Cochin Hand Function Scale, HAQ-DI, and SF-36 physical and mental component.

In contrast, similarly to the general PROs, the majority of organ-specific PROs have not been specifically developed for SSc, but instead for other diseases with the same type of organ involvement.

As an example, the impact of ILD on HRQoL is currently evaluated through questionnaires developed for other lung diseases, such as the Saint George’s Respiratory Questionnaire [157] or the Functional Assessment of Chronic Illness Therapy (FACIT)-Dyspnea questionnaire [158]. These were the two PROs selected as key secondary endpoints in the SENSCIS trial in SSc-ILD [159]. This study pointed out the efficacy of nintedanib in reducing the annual rate of FVC loss, as compared to placebo, although this result was not significantly reflected by these PROs. Noteworthy, both SGRQ and FACIT-Dyspnea are not specifically designed for SSc and consequently, they have not been optimized to take into account or exclude the influence of other disease manifestations that could significantly affect the patients’ experience. Furthermore, according to the inclusion criteria of the SENSCIS trial, the relatively conserved lung function at the baseline (mean FVC %-predicted 72.4 ± 16.8 for the nintedanib group and 72.7 ± 16.6 for the placebo group) has been proposed as a possible explanation for both the relatively low volume loss observed in 12 months and for the lack of impact in the PROs elected as secondary endpoints. This raises the important consideration regarding the asymptomatic phases of some organ involvements in SSc, obviously not translated in the patients’ experience, especially regarding cardio-pulmonary involvement. In fact, these phases potentially represent “the window of opportunity” to treat early organ involvement in order to avoid disease progression to late and irreversible severe stages, finally reflected in the presence of symptoms reported by the patients.

Considering that the direction of future research will be to discover new drugs acting in early SSc, and since patient’s perspective is a crucial determinant for regulatory agencies in defining the overall relevance of a clinical endpoint, future clinical trials should accurately select the key endpoints and correctly integrate the experience of the patients in these early phases of the disease, characterized by relatively conserved functional ability and low impact of the disease on HRQoL.
